# Is COVID-19 Coagulopathy a Thrombotic Microangiopathy? A Prospective, Observational Study

**DOI:** 10.3390/ijms26115395

**Published:** 2025-06-04

**Authors:** Mauro Silingardi, Fulvia Zappulo, Ada Dormi, Attilia Maria Pizzini, Chiara Donadei, Maria Cappuccilli, Chiara Fantoni, Stefania Zaccaroni, Valeria Pizzuti, Nicola Cilloni, Simona Tantillo, Antonella Guidi, Rita Mancini, Gaetano La Manna, Giorgia Comai

**Affiliations:** 1 Department of Internal Medicine, Ospedale Maggiore “Carlo Alberto Pizzardi”, 40133 Bologna, Italy; m.silingardi@ausl.bologna.it (M.S.); attiliamaria.pizzini@ausl.bologna.it (A.M.P.); chiara.fantoni@ausl.bologna.it (C.F.); stefania.zaccaroni@ausl.bologna.it (S.Z.); 2 Nephrology, Dialysis and Kidney Transplant Unit, IRCCS Azienda Ospedaliero-Universitaria di Bologna, 40138 Bologna, Italy; fulvia.zappulo@aosp.bo.it (F.Z.); valeria.pizzuti3@unibo.it (V.P.); giorgia.comai@aosp.bo.it (G.C.); 3 Biostatistics, Department of Medical and Surgical Sciences (DIMEC), Alma Mater Studiorum-University of Bologna, 40138 Bologna, Italy; ada.dormi@unibo.it; 4 Department of Medical and Surgical Sciences (DIMEC), Alma Mater Studiorum-University of Bologna, 40138 Bologna, Italy; chiara.donadei@studio.unibo.it (C.D.); maria.cappuccili@unibo.it (M.C.); 5 Department of Anaesthesia, Intensive Care and EMS, Ospedale Maggiore “Carlo Alberto Pizzardi”, 40133 Bologna, Italy; nicola.cilloni2@unibo.it (N.C.); simona.tantillo@ausl.bologna.it (S.T.); 6 Laboratorio Unico Metropolitano, AUSL Bologna, 40133 Bologna, Italy; guid3@ausl.bologna.it (A.G.); rita.mancini@ausl.bologna.it (R.M.)

**Keywords:** ADAMTS13, complement activation, COVID-19-associated coagulopathy, inflammatory cytokines, von Willebrand factor

## Abstract

Severe COVID-19 is often associated with coagulopathy and thrombotic complications. The underlying mechanisms are complex and multifactorial, involving platelet activation, dysregulation of the complement cascade, fibrinolytic imbalance, release of pro-inflammatory cytokines, immunothrombosis, antiphospholipid antibodies, and alterations in the von Willebrand factor (vWF)/ADAMTS13 axis. These pathways are also implicated in thrombotic microangiopathies (TMAs), characterized by endothelial injury and widespread microvascular thrombosis. In this prospective monocentric observational study, we investigated whether COVID-19-associated coagulopathy meets the criteria for TMA and evaluated the roles of complement activation and vWF/ADAMTS13 imbalance in disease severity. Forty-three hospitalized COVID-19 patients were enrolled and stratified by disease severity. Blood samples collected at admission were analyzed for hematologic, coagulation, inflammatory, and complement parameters. A 30-day follow-up recorded survival and thrombotic events. All patients showed elevated vWF and factor VIII levels; however, only vWF collagen-binding activity (vWF-CBA) significantly correlated with disease severity. ADAMTS13 activity remained above 60% in all cases, and no schistocytes were detected, arguing against a diagnosis of classical TMA. Nevertheless, the vWF-CBA/ADAMTS13 ratio was significantly higher in severe cases, particularly in unvaccinated individuals, suggesting endothelial dysregulation. Complement analysis revealed increased C5a levels and decreased C3b/iC3b ratios in severe disease, consistent with complement activation and consumption. C2 levels were also lower in these patients. Although complement activation and vWF/ADAMTS13 imbalance did not directly correlate, both pathways showed a similar trend according to disease severity. Overall, our findings indicate that COVID-19-related coagulopathy does not fulfill the criteria for classical TMA but shows features of complement-mediated endothelial injury and vWF dysregulation. The vWF-CBA may serve as a rapid, standardized tool for assessing endothelial dysfunction. Activation of the complement system, particularly via the lectin and alternative pathways, appears central to the prothrombotic state in severe COVID-19.

## 1. Introduction

Patients with the severe form of coronavirus disease 2019 (COVID-19) frequently experience coagulation abnormalities. The complex and multifaced pathogenetic link of COVID-19 with the risk of thrombotic events involves several mechanisms, including the activation of platelets, complement system, and coagulation cascade, fibrinolysis, inflammatory cytokines release, immunothrombosis, production of antiphospholipid antibodies, and imbalance of the von Willebrand factor (vWF)/ADAMTS13 axis [1]. Both vWF/ADAMTS13 axis dysfunction and complement system activation have a pivotal role in thrombotic microangiopathies (TMA). This includes a heterogeneous group of diseases characterized by endothelial damage resulting in an increased release of VWF and widespread thrombosis at the microcirculatory level. Diagnostic hallmarks of TMA are consumptive thrombocytopenia and microangiopathic hemolytic anemia (MAHA), with the detection of schistocytes in the blood smear.

Classically, thrombotic thrombocytopenic purpura (TTP) and hemolytic uremic syndrome (HUS) have been classified as primary TMA forms. Secondary forms of TMA can occur in several settings, including autoimmune diseases, cancer, chemotherapy, pregnancy, solid organ transplantation, hematopoietic cell transplantation, and multiple (viral, bacterial, parasitic) infections [2,3].

The possibility of understanding COVID-19-associated coagulopathy as a secondary form of TMA is challenging. Early retrospective studies reported low levels of ADAMTS13 and the presence of schistocytes in the blood smear [4], but there is some inconsistency in currently available data. While some studies found a correlation between low levels of ADAMTS13 and mortality in COVID-19 patients, other reports failed to find such a correlation [5,6,7]. A cross-sectional study reported a proportional increase in the vWF/ADAMTS13 ratio with the severity of the disease [8]. Complement-associated microvascular injury and thrombosis were reported in a retrospective case series [9]. Activation of the complement system was described in a retrospective study on 148 patients; in this study, the level of activation correlates with disease severity [10,11].

Current data have important limitations. Previous studies were mainly retrospective, with no definite inclusion/exclusion criteria, thus suggesting selection bias. This is particularly important considering the wide clinical spectrum of secondary TMA, thus opening to the possibility of overlapping conditions leading to controversial findings. Thus, further studies are needed to better elucidate the role of complement system activation at different levels and degrees in the pathogenesis of TMA [12].

We performed a prospective cohort study in order to clarify the nature of COVID-19 coagulopathy as a possible form of TMA.

## 2. Results

From 26 March to 17 June 2021, a total of 43 patients were enrolled in the study. The patients were categorized into two groups: mild disease (28 patients), requiring any supplemental oxygen or non-invasive ventilation or use of high-flow oxygen devices, and severe disease (15 patients), requiring invasive mechanical ventilation or extracorporeal membrane oxygenation (ECMO). Of 43 patients, 11 (25.5%) patients were not vaccinated: among them, the majority (8/11; 72.7%) were included in the severe group. Ten out of forty-three patients (23.2%) had received a full vaccination schedule (3 boosts/doses).

Table 1 describes the baseline demographic and clinical characteristics of the patients. No statistical differences between the three groups were registered in terms of age, body mass index (BMI), and comorbidity profile.

The two groups did not differ in terms of PT and aPTT and other biochemical variables.

Serum haptoglobin levels were within the normal range in all patients; LDH was slightly increased in both groups, with no significant difference. No schistocytes in the blood smear of any patient were found. The search for antiphospholipid antibodies was negative in all patients. After 30 days, five patients died (all in the severe group). Platelet count was significantly lower in the severe group (*p* = 0.048) (Table 2).

Coagulative tests are detailed in Table 3. An important rise in factor VIII levels was registered in all patients with no significant difference between groups (3.15 vs. 3.14, *p* = 0.646). The same trend was found for fibrinogen, D-dimer, vWAg, and vW-Ricof.

vWF collagen-binding activity (CBA) was instead significantly higher in the severe group of patients (3.44 vs. 3.16, *p* = 0.023). ADAMTS13 activity was well above normal levels in all patients with no significant difference between groups (0.89 vs. 0.80 *p* = 0.093). So far, the vWAg/ADAMTS13 ratio, especially the vWF-CBA/ADAMTS13 ratio, was significantly higher in the severe group. These results were driven by the no-vax population: the chi-square test between vaccinated and not vaccinated groups was highly significant (*p* = 0.004). Three cases of deep vein thrombosis were observed (two in the severe and one in the mild/moderate group). No major bleeding was reported.

The results of complement factor testing are shown in Table 4. At baseline, a significant difference between the mild and severe groups was found in the serum level of C2 and C5a.

**Figure 1:**
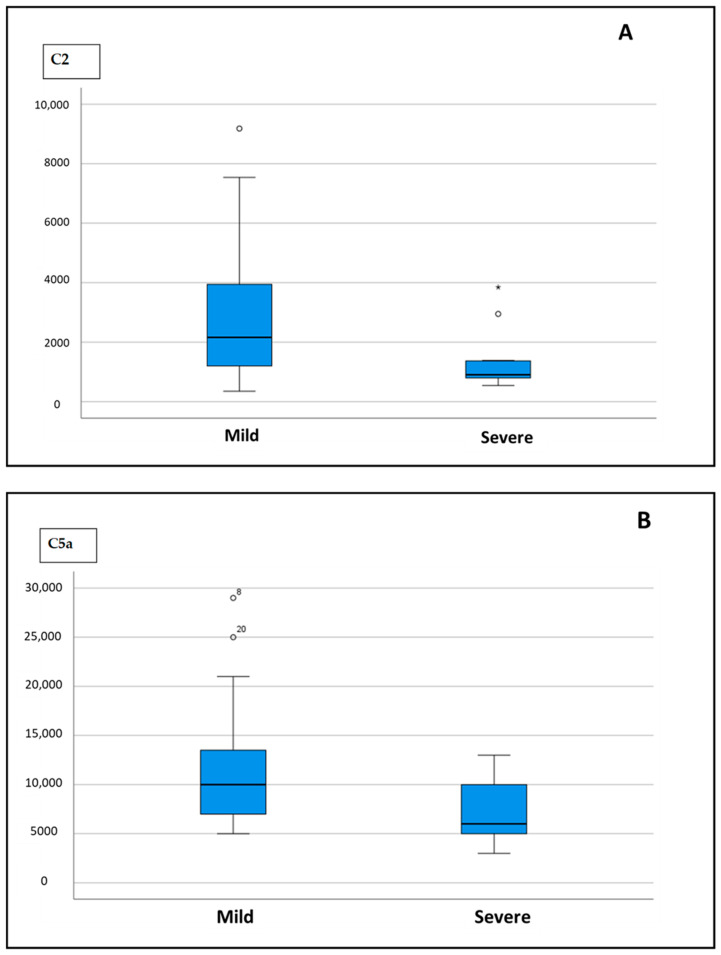
Difference in serum levels of C2 (**A**) and C5a (**B**) in mild vs. severe group.

Serum levels of C2 were significantly lower in the mild than in the severe group (*p* = 0.006), similar to the level of C5a (Figure 1A and 1B, respectively).

**Figure 2:**
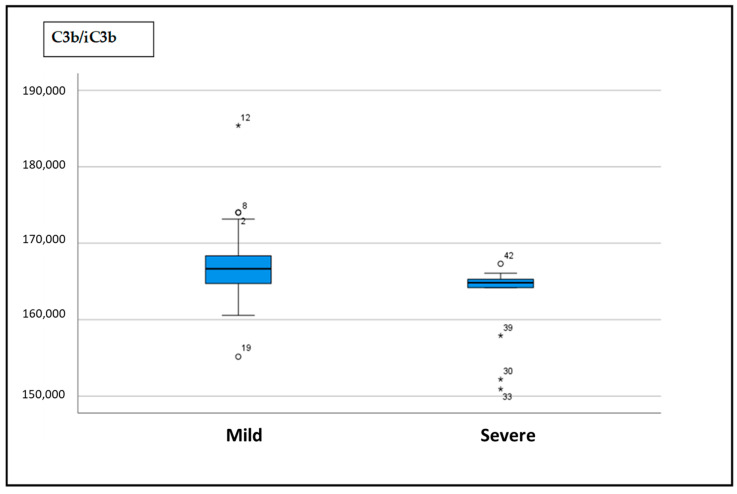
Difference in C3b/iC3b serum levels in mild vs. severe group.

As shown in Figure 2, the C3b/iC3b ratio was significantly lower in the severe group than in the mild group (median 164.84, IQ range 16,106.00–165,380.00, vs. 166.66, IQ range 164,710.00–168,380.00, *p* = 0.037).

## 3. Discussion

This is a monocentric prospective study to investigate the association between COVID-19 and coagulopathy. Moreover, there is an attempt to analyze the involvement of complement in promoting the activation of the coagulation cascade. vWF, the ADAMTS13 axis, and complement activation were evaluated in all patients with mild/moderate and severe COVID-19-associated disease.

An imbalance in the von Willebrand factor (vWF)/ADAMTS13 axis and activation of the complement system occur concomitantly in COVID-19 patients and are significantly more pronounced in those with severe disease. However, we found no evidence of thrombotic microangiopathy (TMA): hemoglobin levels were comparable between groups, schistocytes were not observed on peripheral blood smears, and serum haptoglobin levels remained within the normal range. The only notable hematologic abnormality was a significant reduction in platelet count. Lactate dehydrogenase (LDH) levels were mildly elevated but did not differ significantly between groups, and ADAMTS13 activity remained above 60% in all patients.

To our knowledge, this is the first methodologically rigorous, prospectively designed study specifically aimed at evaluating COVID-19-associated coagulopathy as a form of secondary TMA. Several earlier prospective studies conducted during the initial phase of the pandemic [13,14,15,16,17,18,19] investigated either the vWF/ADAMTS13 imbalance or complement activation as prognostic markers for hospitalization or in-hospital mortality. However, these studies lacked specific exclusion criteria for secondary TMA and did not report simultaneous measurements of both the vWF/ADAMTS13 axis and complement activity.

Our study, conducted during the second wave of the pandemic, enrolled a demographically and clinically distinct cohort that included both vaccinated and unvaccinated individuals—unlike previous studies, which predominantly included treatment-naïve patients. We observed significantly lower vWF/ADAMTS13 ratios (2.73 in mild/moderate cases vs. 4.40 in severe cases) compared to those reported in the retrospective study by Mancini [8]. Several factors may explain this discrepancy: the dominance of the Delta variant, known for its distinct pathogenic profile [9], and the implementation of standardized treatments, including dexamethasone and prophylactic low-molecular-weight heparin (LMWH). Moreover, the prospective design of our study allowed for a more consistent temporal evaluation of laboratory parameters compared to cross-sectional approaches.

Unvaccinated patients exhibited a more pronounced vWF/ADAMTS13 imbalance, suggesting a potentially higher thrombotic risk. This observation is consistent with recent studies demonstrating increased thromboembolic events and pulmonary embolism in unvaccinated individuals, pointing toward a possible protective effect of vaccination (1). In a separate single-center retrospective analysis, we also identified a significantly increased risk of pulmonary embolism among unvaccinated COVID-19 patients admitted to both intensive care and general medical wards [20].

Importantly, vWF collagen-binding activity (vWF-CBA), rather than vWF antigen (vWF:Ag) or ristocetin cofactor activity (vWF:RCo), showed a significant correlation with COVID-19 disease severity [21,22]. vWF-CBA serves as a rapid, indirect surrogate for high-molecular-weight vWF multimers (HMvWF). Although the presence of ultra-large vWF (ULvWF) multimers has not been directly demonstrated in COVID-19 patients, it is widely accepted that an excess of HMvWF relative to normal plasma promotes a prothrombotic state [11]. Our findings further support the concept of COVID-19 as an endotheliopathy and highlight the potential of vWF-CBA as a readily accessible biomarker of endothelial injury and thrombotic risk.

In the second part of our study, we investigated complement activation and its association with COVID-19 severity. Severe cases showed increased C5a levels and reduced C2 and C3b/iC3b, indicating complement overactivation and consumption.

Several studies have explored this mechanism. Yu et al. proposed the C3a/C3 ratio as a marker of complement consumption [23]. Gao et al. demonstrated that the SARS-CoV-2 nucleocapsid protein binds mannose-binding lectin (MBL), activating MASP-2 and the lectin pathway, leading to C4 cleavage and complement deposition [6]. The N330 glycosylation site on the spike protein plays a key role in MBL binding. More recent data suggest that SARS-CoV-2 also activates MASP-1 and MASP-3, possibly triggering the alternative pathway [19]. Increased MBL and ficolin expression (e.g., FCN-3) in COVID-19 further support lectin pathway activation [24].

Data from the RCI-COVID-19 group showed elevated C3a, sC5b-9, and C3c in severe cases. Low serum C3 and high C3a/C3 ratios were independently associated with poor outcomes, even after adjustment for confounders [25].

Autopsies revealed microvascular thrombosis with the deposition of complement components (C5b-9, C4d, MASP-2) in organs such as the lungs and skin, supporting complement-driven endothelial damage [26]. Bilgin et al. defined this as Inflammatory Thrombosis with Immune Endotheliitis (ITIE) [27].

Endothelial injury further promotes coagulation via VEGF and PDGF release [28]. While we found no direct link between complement activation and the vWF/ADAMTS13 axis, experimental models suggest that high-molecular-weight vWF may act as a complement cofactor [29,30].

**Figure 3:**
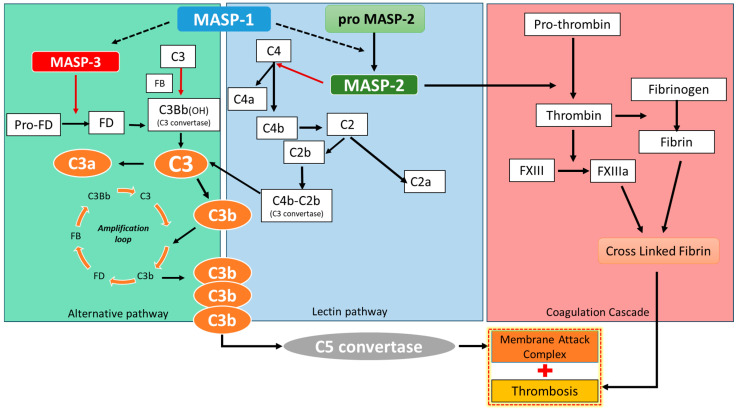
**Cross interaction between the complement system and coagulation cascade.** Complement pathways in SARS-CoV-2 infection. Activation of the lectin pathway by the virus via the MBL/MASP-1/MASP-2 complex runs out after binding of MBL/MASP complexes to the surface of pathogens. MASP-1 autoactivates and transactivates MASP-2, and C2 and C4 components are cleaved generating the C3 convertase. The alternative pathway is initiated by the spontaneous hydrolysis of component C3, generating C3a and C3b. C3b binds to factor B and is cleaved by factor D, forming the C3 convertase of the alternative pathway. After this step, the three pathways converge into a single pathway. The C3 convertase enzyme cleaves component C3 into C3a and C3b. C3a and C4a are anaphylatoxins that contribute to an increase in inflammatory processes and to the chemotaxis of neutrophils and macrophages. MASP-3 also participates in the cleavage of pro-factor D into factor D of the alternative pathway. The elements of the figure are not shown in their actual proportions. MASP activation participates in coagulation cascade activation. MASP-2 determines pro-thrombin cleavage in thrombin that promotes coagulation activation.

Finally, complement activation contributes to early and late phases of coagulopathy: MASP-1, MASP-2, and thrombin promote D-dimer elevation, thrombocytopenia, and prolonged PT in early disease, while later MASP-1-driven activation of prothrombin and fibrinogen leads to widespread thrombosis and fibrin degradation [31]. The complex interaction between complement activation, the vWf–ADAMTS13 axis activation and the coagulation cascade is described in Figure 3 (Figure 3).

Our findings highlight the simultaneous activation of both the vWF/ADAMTS13 axis and the complement system in severe COVID-19 cases. The contemporary activation of complement may reflect a shared upstream trigger, such as widespread endothelial injury, or indicate reciprocal amplification between the two pathways. Notably, the presence of high-molecular-weight vWF multimers (HMvWF), known for their strong prothrombotic potential, may either enhance complement activation or represent a surrogate marker of the same inflammatory-endothelial cascade. Experimental models suggest that HMvWF can act as a cofactor in complement activation, reinforcing the hypothesis of a pathogenic link between endothelial damage, vWF multimer accumulation, and complement-mediated injury.

## 4. Material and Methods

### 4.1. Study Design and Population

This is a prospective single-center observational study on hospitalized patients in the Internal Medicine of Maggiore Hospital in Bologna. Patients with COVID-19 in Italy from 26 March to 17 June 2021 were considered.

The primary endpoint was to assess if the simultaneous activation of vWF/ADAMTS13 and complement plays a role in the pathogenesis of COVID-19-associated coagulopathy and its impact on the patient’s outcome.

The secondary endpoints were (a) to describe the clinical and laboratory features of COVID-19-associated coagulopathy and find laboratory signs of thrombotic microangiopathy and (b) to analyze the patterns of complement activation in COVID-19-associated coagulopathy.

Inclusion criteria were as follows: (a) age > 18 years; (b) COVID-19-related hospitalization with positive nasopharyngeal swab and bilateral interstitial pneumonia documented by high-resolution computer tomography (HRCT).

Exclusion criteria were as follows: (a) pregnancy and lactation; (b) bacterial sepsis; (c) concomitant drugs as a possible cause of thrombotic microangiopathy (chinidine, gemcitabine, oxaliplatin); (d) malignant hypertension; (e) autoimmune diseases; (f) solid organ transplantation; (g) hematopoietic stem cell transplantation; (h) active cancer; (i) major surgery within 30 days before; and (j) Major Adverse Cardiovascular Events (MACE) within 30 days before.

The patients were admitted both in the Internal Medicine Ward and ICU. They were treated according to WHO guidelines during the COVID-19 Delta wave, in particular, using steroids and low-molecular-weight heparin (LMWH) as prophylactic dose. Informed consent was obtained from all participants. For each patient, blood samples were collected at admission, and a complete blood count was performed. Biochemical variables such as haptoglobin, fibrinogen, LDH, and D-dimers were also assayed. A list of all the tests performed is reported in Table 5.

After 30 days, a clinical (or telephone) follow-up was scheduled in order to assess for survival and thrombotic (arterial/venous) or bleeding complications, i.e., major bleeding, according to the classification of the International Society on Thrombosis and Haemostasis (ISTH) [13].

The study was carried out in accordance with the principles of the Declaration of Helsinki and approved by the AVEC Ethics Committee, Azienda Ospedaliero-Universitaria di Bologna, n° 179-2021-OSS-AUSLBO (date of approval 15 February 2021). An informed consent form was distributed to all participants and signed.

### 4.2. Laboratory Features

#### 4.2.1. Blood Assays

White blood cells (WBC), hemoglobin (Hb), and platelets (PLT) were determined on whole blood samples collected in K3EDTA tubes (Vacutainer, Becton Dickinson, Broken Bow, NE, USA) using an automated hematology analyzer Sysmex XN-Series (distributed by Dasit Group Spa, Cornaredo, Italy). The count of schistocytes was performed and expressed as a percentage (on 1000 red cells) using an optical microscope on the peripheral blood smear generated by an automated slidemaker stainer with the May-Grunwald Giemsa stain.

Ferritin and procalcitonin were determined on serum samples by the immunoturbidimetric method on UniCel DxI (Beckman Coulter Inc., Ireland, CA, USA). Haptoglobin, lactate dehydrogenase (LDH), C-reactive protein (CRP) and serum creatinine was assayed using the analyzer AU Series (Beckman Coulter Inc., Ireland, CA, USA). Interleukin-6 was determined on a serum sample by an Immulite 2000 immunoassay system (Medical Systems Spa, Genova, Italy).

#### 4.2.2. Coagulation

Coagulation tests, namely ADAMTS13, factor VIII, antithrombin, fibrinogen, D-dimers, VWF:Ag, VWF:CBA, VWF:RCO, and lupus anticoagulant, were performed in plasma samples. Briefly, blood samples were collected into 0.109 mol/L sodium citrate tubes (Vacutainer, Becton Dickinson, Broken Bow, NE, USA) and centrifuged at 2000× *g* for 15 min at room temperature. Samples for lupus anticoagulant tests were centrifuged twice, and the second centrifugation was performed at 2500× *g* for 10 min at room temperature. All samples were frozen at −80 °C within 4 h of collection and stored until analysis.

IgG/IgM aCL and IgG/IgM anti-β2GPI were determined on serum samples using fluorescence enzyme immunoassay (FEIA) (EliATM, Phadia AB, Uppsala, Sweden).

A VWF:Ag test was performed using the vWF Ag assay (Siemens Healthcare Diagnostics Products GmbH, Marburg, Germany). VWF ristocetin cofactor assay (VWF:RCo) was performed using the VWF:GPIbR method with BC von Willebrand reagent (Siemens Healthcare Diagnostics, Erlanger, Germany). FVIII was measured with a chromogenic method using the Factor VIII Chromogenic Assay (Siemens Healthcare Diagnostics). Fibrinogen and antithrombin were measured using Dade Thrombin reagent and Innovance Antithrombin, respectively (Siemens Healthcare Diagnostics). D-Dimers were determined as an agglutination assay using Innovance D-Dimer (Siemens Healthcare Diagnostics Products, Erlangen, Germany). Lupus anticoagulant autoantibodies were measured through two different screening and mixing tests: diluted Russell’s Viper Venom Time (dRVVT) with LA1 screening reagent (Siemens Healthcare Diagnostics, Erlangen, Germany) and an aPTT-based test with PTT-LA (Stagò Diagnostic S.A.S., Asnières sur Seine Cedex, France). Confirmation tests for dRVVT and aPTT-based tests were performed using LA2 confirmation reagent (Siemens Healthcare Diagnostics, Enlangern, Germany) and PTT-LA with lyophilized platelets (Biodata Corporation, Horsham, PA, USA), respectively. All these tests were performed on the Sysmex CS-5100 analyzer (distributed by Siemens Healthineers, Forchheim, Germany).

ADAMTS13 activity and VWF:CB were measured simultaneously on an ACL Acustar analyzer with the HemosIL ADAMTS13 Activity assay and HemosIL VWF:CB assay, respectively (Instrumentation Laboratory, Bedford, MA, USA). The HemosIL AcuStar ADAMTS13 Activity assay is a fully automated, two-step CLIA based on the chemiluminescent detection of the fragments generated by ADAMTS13. The HemosIL VWF:CB assay uses magnetic beads coated with a type III collagen triple-helical peptide.

#### 4.2.3. Complement Proteins

The simultaneous quantification of the complement proteins was performed in plasma using Luminex xMAP technology. Complement system proteins (C2, C4b, C5, C5a, complement factor I, factor D adipsin, C1q, C3, C3b/iC3b ratio, C4, factor B, factor H) were evaluated for each patient at admission.

Following blood centrifugation, plasma samples were collected and stored at −80 °C until subsequent analysis. Samples were thawed at room temperature and complement proteins were quantified using the MILLIPLEX Human Complement Panel 1 and Panel 2 (Table 1) (Cat. No. HCMP1MAG-19K and HCMP2MAG-19K, Merck KGaA, Burlington, MA, USA) according to the manufacturer protocol, and the concentration of each analyte was measured by MAGPIX TM (Luminex^®^ xMAP^®^ Technology, Austin, TX, USA). All samples were analyzed in technical triplicates.

Laboratory tests are summarized in Table 5. 

### 4.3. Statistical Analysis

Sample size was calculated considering the incidence of hospitalization in the ICU and internal medicine operating unit of the Maggiore Hospital in Bologna. Due to the characteristics of the pathology, the incidence is highly variable, so the “Cohort Study with No background incidence” method was used to calculate the sample, using the average proportion of patients accessing the Ospedale of Maggiore with respect to the Italian population reported by the Istituto Superiore di Sanità. Assuming a representative power of the study of 80%, an α error of 5%, and a dropout rate of 20%, the final study sample is estimated at 42 patients.

The normality of the distribution of the data was examined with the Kolmogorov–Smirnov test. Continuous variables are expressed as mean ± SD or median with an interquartile range as appropriate, and categorical variables are expressed as a percentage. For non-normally distributed variables, comparisons between two groups were made using the U Mann–Whitney test for independent samples. The Wilcoxon test was used for paired data, while 3 or more groups were compared with the Kruskal–Wallis test. Categorical variables were evaluated by a chi-square test or Fisher test, as appropriate. The correlation between variables was analyzed through the Spearman test. A *p* value < 0.05 was set as the threshold for statistical significance, and all the analyses were conducted on SPSS version 28 [SPSS Inc., Chicago, IL, USA], Microsoft Windows version.

## 5. Conclusions

Our findings highlight the simultaneous activation of both the vWF/ADAMTS13 axis and the complement system in severe COVID-19 cases. The dedicated, prospective design to investigate the role of the vWF/ADAMTS13 axis and complement activation, in order to clarify the issue of COVID-19 coagulopathy as a possible secondary TMA, is the main strength of our study. Strict inclusion/exclusion criteria and consecutive hospitalized patients minimized possible selection bias. The demonstration of both activated pathways, even in the absence of signs of TMA, could enhance the hypothesis that the complement activation and vWF/ADAMTS13 axis both contribute to the pathogenesis of COVID-19 coagulopathy.

## Figures and Tables

**Table 1:** Baseline characteristics of patients.

	Mild (*n* = 28)	Severe (*n* = 15)	*p*
Male sex, %	71.40%	73.30%	0.594
Age (years), median (IQR)	62.00 (49.25–69.50)	61.00 (53.00–68.75)	0.894
BMI (kg/m^2^), median (IQR)	26.75 (25.00–30.98)	29.40 (36.50–36.00)	0.157
Diabetes, n (%)	6 (30%)	2 (18.20%)	0.394
Hypertension, n (%)	9 (45%)	7 (63.60%)	0.269

**Table 2:** Evaluation of biochemical and hematological variables.

	Mild (n = 28)	Severe (n = 15)	*p* *
WBC (10^9^/L)	8.4750 (6.395–10.665)	8.690 (5.740–12.230)	0.819
Hb (gr/L)	12.9 (11.63–13.70)	12.1 (11.60–12.80)	0.296
PLT count (10^9^/L)	292.50 (235.75–382.00)	248 (197–285.00)	**0.048**
Haptoglobin (mg/dL)	274.50 (196.25–354.75)	289.50 (173.50–371.25)	0.915
LDH (UI/L)	340.00 (258.00–403.00)	357.00 (327.00–499.00)	0.860
D-dimer (mg/L FEU)	0.70 (0.41–1.63)	1.87 (0.76–3.57)	**0.041**
Fibrinogen (mg/dL)	458.00 (314.00–554.00)	467.00 (370.00–605.00)	0.397
AT (%)	103.50 (95.2–117.00)	108.00 (91.00–119.00)	0.855
Ferritin (ng/mL)	648.00 (241.00–963.00)	944.00 (330.00–1854.00)	0.317
CRP (mg/dL)	2.58 (1.25–9.79)	4.01 (0.99–14.72)	0.449
IL-6 (pg/mL)	15.30 (6.65–155.18)	31.05 (11.70–82.88)	0.510
PCT (ng/mL)	0.05 (0.05–0.20)	0.05 (0.05–0.53)	0.375
Serum creatinine (mg/dL)	0.76 (0.64–0.92)	0.80 (0.67–1.45)	0.629

* significant *p* values are highlighted in bold. Abbreviations: WBC, white blood cells; Hb, hemoglobin; PLT, platelets; LDH, lactate dehydrogenase; AT, antithrombin; CRP, C-reactive protein; IL-6, interleukin 6; PCT, procalcitonin.

**Table 3:** Coagulation test results.

	Mild (n = 28)	Severe (n = 15)	*p* *
vWF:CBA (UI/mL)	3.16 (2.24–3.64)	3.44 (3.13–4.90)	**0.023**
ADAMTS 13 (UI/mL)	0.89 (0.82–1.14)	0.80 (0.70–1.07)	0.093
vWF:RICOF (UI/mL)	2.40 (1.79–3.57)	3.65 (2.30–3.82)	0.169
vW:AG (UI/mL)	2.88 (1.94–3.78)	3.65 (2.30–4.11)	0.090
Factor VIII (UI/mL)	3.15 (2.50–3.51)	3.14 (2.58–3.82)	0.646

* significant *p* values are highlighted in bold. Abbreviations: vWF:CBA, von Willebrand factor collagen-binding activity; ADAMTS13, ADAM metallopeptidase with thrombospondin type 1 motif 13; vWF:RICOF, von Willebrand factor ristocetin cofactor activity; vW:AG, von Willebrand factor antigen; ACA, anticardiolipin; GPI, glycoprotein 1.

**Table 4:** Results of complement protein assay.

	Mild (n = 28)	Severe (n = 15)	*p* *
C2	2161.11 (185.700–3947.700)	904.70 (789.800–1370.400)	**0.006**
C4b	9930.50 (5545.550–12,996.850)	8794.49 (7043.900–23,535.800)	0.999
C5	16,904.30 (13,078.550–24,302.050)	16,164.80 (12,696.900–35,980.000)	0.962
C5a	10,000 (7000–13,750)	6000 (4925–10,500)	**0.015**
CFI	43,946.30 (29,112.000–55,291.700)	32,804.20 (25,427.700–60,340.800)	0.445
MBL	2428.60 (2004.050–4868.900)	3811.00 (2693.600–5010.800)	0.176
C1q	22,840.00 (19,800.00–25,740.00)	28,880.00 (23,080.00–31,440.00)	0.860
C3	22,360 (14,150.00–32,450.00)	25,560.00 (12,680.00–34,100.00)	0.703
C3b/iC3b	166,660.00 (164,710.000–163,830.00)	164,840.000 (161,060.00–165,380.00)	**0.037**
C4	537,400.00 (345,250.00–746,300.00)	759,720.00 (415,460.00–861,680.00)	0.192
Factor B	232,840.00 (164,910.00–276,460.00)	247,880.00 (197,840.00–294,389.00)	0.611
Factor H	253,000.00 (212,510.00–314,790.00)	304,480.00 (257,300.00–353,460.00)	0.086

* significant *p* values are highlighted in bold. Abbreviations: Complement factor 2, C2; complement factor 4b, C4b; complement factor 5, C5; complement factor 5a, C5a; adipsin mannose-binding lectin, MBL; complement factor I, Factor I; complement 1q, C1q; complement factor 3, C3; complement factor C3b/iC3b, C3b/iC3b; complement factor 4, C4; complement factor B, Factor B; complement factor, Factor H.

**Table 5:** List of tests for the analysis of blood samples.

Hematology	Biochemistry	Coagulation Tests	Complement
White blood cells (WBC)	Lactate dehydrogenase (LDH)	von Willebrand factor collagen-binding activity (vWF:CBA)	Complement C2 (C2)
Hemoglobin (Hb)	Creatinine (Cr)	ADAM metallopeptidase with thrombospondin type 1 motif 13 (ADAMTS 13)	Complement C4b (C4b)
Platelets (PLT)	C-reactive protein (CRP)	von Willebrand factor ristocetin cofactor activity (vWF:RICOF)	Complement C5 (C5)
	Procalcitonin (PCT)	von Willebrand factor antigen (vW:AG)	Complement C5a (C5a)
	Interleukin 6 (Il-6)	Anticardiolipin IgM and IgG (IgM ACA, IgG ACA)	Adipsin mannose-binding lectin (MBL)
	Aptoglobin	Anti-β2-Glycoprotein IgM (Anti-β2-GPI IgM)	Complement factor I (Factor I)
	Ferritin	Anti-β2-Glycoprotein IgG (Anti-β2-GPI IgG)	Complement C1q (C1q)
	Fibrinogen		Complement C3 (C3)
			Complement C3b/iC3b (C3b/iC3b)
			Complement C4 (C4)
			Complement factor B (Factor B)
			Complement factor H (Factor H)

## Data Availability

The datasets used and/or analyzed during the current study are available from the corresponding author on reasonable request.
